# BOX38, a DNA Marker for Selection of Essential Oil Yield of *Rosa × rugosa*

**DOI:** 10.3390/biom13030439

**Published:** 2023-02-25

**Authors:** Jianwen Wang, Yue Liang, Yadong Chu, Liguo Feng

**Affiliations:** College of Horticulture and Landscape Architecture, Yangzhou University, Yangzhou 225009, China

**Keywords:** *Rosa rugosa* Thunb, Box38, repeat number, essential oil

## Abstract

*Rosa rugosa* L. was a famous aromatic plant whose cultivars (*Rosa × rugosa)* have been widely used in the perfume industry in Asia. The perfume market looks for rose cultivars bearing higher essential oil, while the oil yields of most *R. × rugosa* have not been evaluated due to limiting conditions, such as insufficient cultivation areas. Here, we tested the yield and the aroma components of essential oil of 19 *R. × rugosa.* The results indicated that the yields of nerol, citronellol, and geraniol could represent an alternative index of the total yield of essential oil. Sequence syntenic analysis indicated that the *Rosa* genus specific cis-element Box38 was highly polymorphic. The Box38 region isolation of *Rosa × rugosa* by flanked primers proved that Box38 repeat number was significantly positively correlated with the essential oil yield of the corresponding cultivar. In the breeding of *Rosa × rugosa*, six-Box38-repeat could be a robust threshold for selection of high-essential-oil roses. Together, we found that Box38 was a DNA marker for essential oil yield and that it would be helpful in the early selection and breeding of essential oil roses.

## 1. Introduction

Essential oil plants are favored in industrial crop cultivation due to the great value of their essential oils for food preservation [1], aromatherapy [2], medicine [3], and flavors [4]. The perfume rose is an ancient essential oil plant and is famous for its rose essential oil. In about 200 species of *Rosa* genus, only dozens of species with a strong fragrance were noticed particularly by humans, e.g., *Rosa chinensis* cv. ‘Old Blush’ (Old Blush), *Rosa rugosa* (Rugosa, *R. rugosa*), *Rosa damascena, Rosa centifolia*, and *Rosa alba* [5,6]. These fragrant species have been widely used in the breeding of the perfume roses [7]. The Middle East (Damascus) origin *R. damascena* and its hybrid cultivars *(R. × damascena)* contribute mostly to the Europe market [8]. For example, *R. × damascena* ‘trigintipetala’, a famous oil bearing rose, has been planted in Turkey and Bulgaria since the 16th century and was introduced to Asia (China) in the 1970s [9]. Meanwhile, the East Asia (China and Japan) origin *R. rugosa* and its hybrid cultivars *R. × rugosa* are more popular in the Asian market [10,11].

Plant essential oil composition is related with many factors, e.g., geographical area of production [12,13]; harvest year [13]; irrigation [14]; extraction system [15]. For the essential oil production of *R. × rugosa*, unified methods including harvest timing (3- to 5-year-old plants), hydrodistillation extraction and open field culture without irrigation indicated that only geographical area and cultivar differences should be the key factors. The abundant cultivars of *R. × rugosa* came from the allele recombination of several wild species and spontaneous bud mutations of cultivars, e.g., the national geographic indication cultivar of China, *R. × rugosa* ‘Kushui’, planted in Kushui (Gansu Province, China) since the 18th century, is a natural hybrid of *Rosa setate × R. rugosa* [10,16]. Another China geographic indication cultivar, *R. × rugosa* ‘plena’ (Plena)*,* planted in Pinyin (Shandong Province, China) since the 16th century, is a spontaneous variety of wild *R. rugosa* [17]. In addition to the above several famous cultivars, some oil roses were bred or introduced to China since the 1970s. Most of these oil roses lacked a paternal genetic background due to the mixed pollination in breeding processes, and their oil yield needed to be evaluated urgently [18]. Considering the high cost and long period of commercial planting, the fast and low-cost method based on genetic markers should be an executable solution [7,19,20].

Geraniol is the key index of commercial rose essential oils. Most plants convert geranyl diphosphate (GPP) to geraniol by a plastid monoterpene synthase [21]. While modern roses rely on a specific cytosolic pathway [22,23] which dephosphorylates GPP to geranyl phosphate (GP) by Nudix hydrolase (*NUDX1*), then to geraniol by one uncharacterized phosphatase. The *NUDX1* family includes 4 sub-family, i.e., Nudx1-1(a/b), −2, −3, −4, while only NUDX1-1a gene clusters are specific to geraniol producing rose species whose NUDX1-1a copy number is more than non-geraniol-producing species [24,25]. The NUDX1-1a copy number positively correlated with the NUDX1-1a expression, and it could be a candidate marker for high geraniol level cultivars or mutations [24]. Besides the NUDX1-1a gene, transposon elements (TEs) in promotors could also be candidate markers for scented-rose breeding [21,24]. Here, based on the analysis of the conservation and variation of transposon fragments relevant motifs in *R. × rugosa*, a rose-specific motif, Box38, was selected as a robust marker for the early selection of high-oil-yield cultivars.

## 2. Materials and Methods

### 2.1. The Rose Cultivars and Sampling

Samples were collected from the rose germplasm resource nursery (116.457676° E, 36.288978° N) of Pingyin Rose Research Institute (Shandong province, China). All 19 *R. × rugosa* cultivars and their accession name used in this study were listed in Supplementary Table S1. The plants were cultivated in the open air under natural climate (temperate monsoon climate) with no fertilizers. Over 15 g leaves of each four-year-old plant were collected for three biological replicates and frozen in liquid nitrogen for DNA extraction. At the same time, the fresh flowers of were picked before 6:00 am (before sunrise) in May–July of 2011 and used for oil extraction instantly. The follower collection and oil extraction were repeated in May of 2022 except for several continuous-flowering cultivars.

### 2.2. Hydrodistillation Extraction and Gas Chromatography with Mass Spectrometry (GC-MS) Analyses of Essential Oil

At least 500 g flowers were distilled with deionized water (4 mL water per gram flower) at 240 °C for 0.5 h and at 180 °C for 1.5 h using a Clevenger-type apparatus. Then, the volatile oil layer settling on top of the aqueous layer were collected. The water in rough extractions was absorbed by anhydrous sodium sulfate, and then essential oils were prepared. The essential oils were diluted by n-hexane to the appropriate concentration (*v*/*v* = 1:1000). For GC-MS analysis, 50 μL dissolved essential oils and 50 μL 3-nonanone (internal standard) were added to the sample injector of a Trace DSQ GC-MS (Thermo Corporation, Waltham, MA, USA). The chromatographic column (ECONO-CAP, EC-1000, Alltech Corporation, Lexington, KY, USA) at the 1.00 mL/min helium flow rate was programmed as follows: 50 °C for 1 min (initial temperature), then increased to 140 °C at 10 °C/min for 5 min, and finally increased to 210 °C at 4 °C/min for 15 min. Then, 1 μL of sample was injected at a separation ratio of 100:1 and injector temperature of 240 °C. The mass spectral ionization temperature, 200 °C; automatic scanning at *m*/*z* 50–550 amu. The electron energy was 70 eV. Qualitative analysis based on NIST17 MS database and quantitative analysis based on peak areas normalized by internal standard were performed according to our previous methods [26].

### 2.3. Mapping and Syntenic Analysis of NUDX1-1 Cluster

The genomes of Rugosa and Oldbush were obtained from GDR (Genome Database for Rosaceae, https://www.rosaceae.org/, accessed on 10 January 2022). The 2 haplotype genomes of Zizhi were assembled by our lab (unpublished), namely the first haplotype of Zizhi (Zizhi_H1) and the second haplotype of Zizhi (Zizhi_H2). The homologous gene pairs within the 4 genomes were identified by BLASTP search (*E* < 10^−5^, top five matches). Based on the location of homologous pairs, MCScanX [27] identified the syntenic regions. With the reference of NUDX1-1a cluster of Oldbush, syntenic regions of NUDX1-1a clusters of Rugosa, Zizhi_H1, and Zizhi_H2 were illustrated using BLASTN search (*E* < 10^−5^). Then, the candidate NUDX1-1a genes of the syntenic regions were checked for the complete coding sequence (CDS). Other elements, e.g., Box38 (B38), TEs like Copia R24588 (class I retrotransposon), and Miniature Interspersed TEs (MITEs) P580.2030/G13554 (TE ID in GDR: noCat_denovoRcHm_v2.0-B-P580.2030-Map20, and DXX-MITE_denovoRcHm_v2.0-B-G13554-Map6), were identified by sequence aliment with software Geneious [28].

### 2.4. Distinguish of B38 Copy Numbers by Length of PCR-Products

DNAs of all cultivars were isolated from corresponding leaves using a DNAprep Pure plant kit (Tiagen, China) following the manufacturer’s recommended instructions. Cloning of B38 repeat region was performed with PCR amplification using high fidelity polymerase PrimeSTAR (Takara, Japan). The PCR parameters with primers (5′-TTTGCAAGAAACTAATGCTG-3′ and 5′-GTTACGAATTATTACAAATA-3′) were as follows: 98 °C for 1 min, 25 cycles of (98 °C for 5 s, 58 °C for 5  s, and 72 °C for 10  s), and 72 °C for 1 min. Then, the length of PCR products was distinguished by the 6% polyacrylamide gel electrophoresis (PAGE). The two primers were located on the upstream sequence of the first Box38 repeat and the downstream sequence of the last repeat. The expected PCR products would be 40-bp longer than the Box38 repeat region.

## 3. Results

### 3.1. The Alternative Index of Essential Oil Yield

Most commercial products of rose essential oil were concocted by nature plant flowers based on water-steam distillation. We isolated essential oils by the traditional method to detect the oil yield (Table S1). In the 19 cultivars, Plena_alba (0.4162 μL/g), Russia, Fenghua, and Pingyin were the top four whose oil yields exceeded 0.3 μL/g (0.3 μL oil per gram flowers). The oil yields of six cultivars, i.e., Tuwei, Kushui, HENSA, Daguo, Zizhi_DH and Zizhi (0.0314 μL/g), were inferior to 1 μL/g and others ranged from 0.1 μL/g to 0.3 μL/g. The highest oil yield (0.4162 μL/g), lowest oil yield (0.0314 μL/g), and variable coefficient 58.36% indicated the significantly differential oil yield among these cultivars.

**Figure 1:**
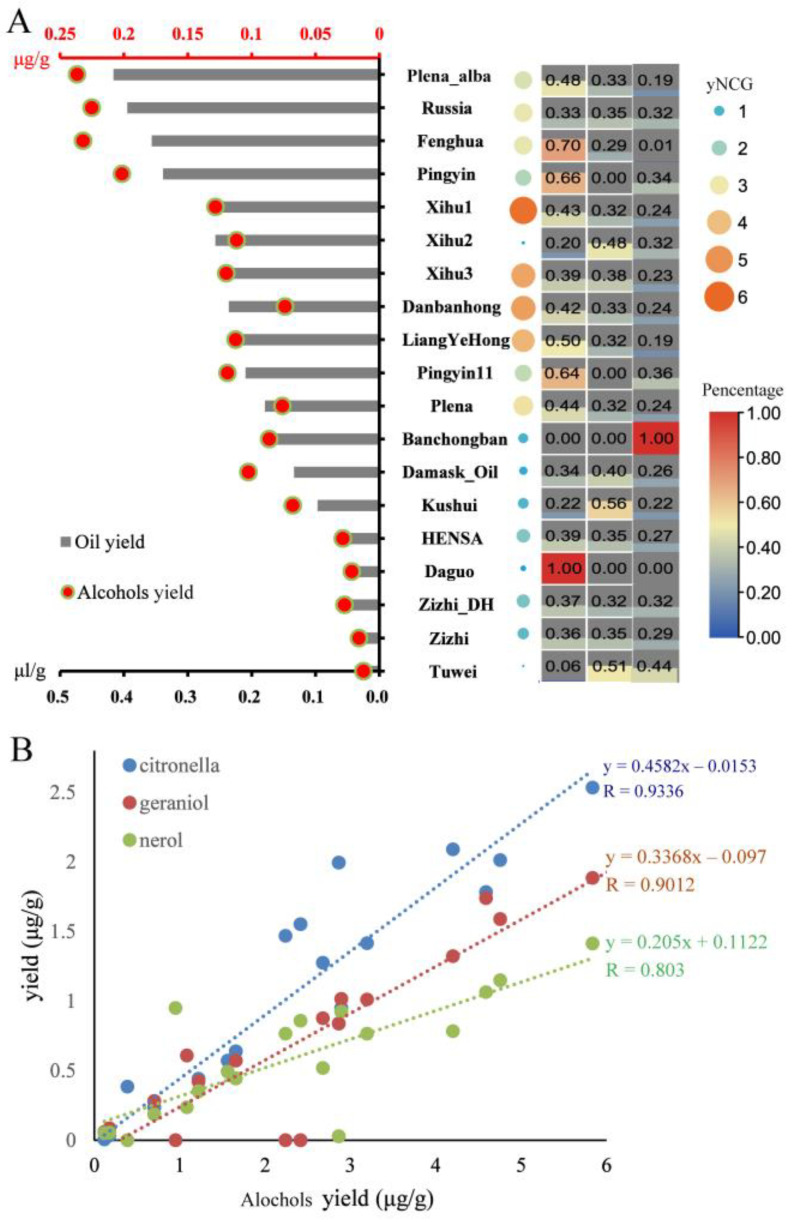
The yNCG (yields of nerol, citronellol and geraniol) was an alternative index of essential oil yield of oil bearing roses. (**A**) The oil yield (histogram), alcohols yield (scatter diagram), yNCG (heatmap, colors and areas of circles) and percentage of each compound (heat map, coloring areas and numbers in the boxes) of 19 rose cultivars. (**B**) Correlation between the alcohols yield and the total yields of nerol, citronellol and geraniol.

GC-MS analysis of essential oils indicated that compared with the low content compounds (arenes, ketones, aldehydes, acids), hydroxy compounds (or alcohols), alkanes and esters were the top 3 compounds contributing to 31–76%, 7–31% and 0.27–32% of oil contents, respectively (Table S2). The alcohols yield was positively correlated with oil yield (Figure 1A). Among the alcohols, we selected nerol, citronellol, and geraniol as the key compounds of essential oils since they were much richer than other minor compounds, e.g., linalool, diphenyl ethanol, farnesol, and bisabolol (Tables S3 and S4). Moreover, the yields of nerol, citronellol, and geraniol (yNCG) were significantly positively correlated with alcohol yield (Figure 1B and Table S4). yNCG would be an alternative index of essential oil yield. Xihu1, Xihu3, Dnabanhong, and Linagyehong were richest in the yNCG. All three compounds contributed to the yNCG, except Banchongban, Daguo, and Pingyin (Figure 1A).

### 3.2. Cosserved NUDX1-1a Clusters and Variable B38

**Figure 2:**
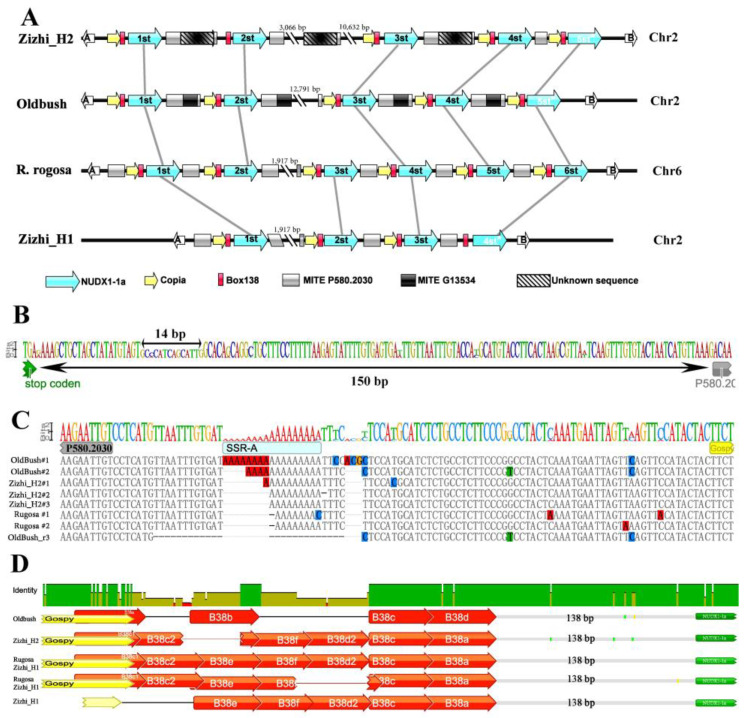
Gene map and micro-synteny of NUDX1-1a clusters and sequence aliments of conserved elements. (**A**) Gene copies and shared elements indicated by 1st–6st were based on the systematic compare of homologous 2st chromosomes of Oldbush. The synteny distances between genes and elements were approximative and long sequences (with on NUDX gene, double dashed) were distorted to show the relative organization. Chr, chromosome. Large blue arrows, NUDX1-1a genes; large yellow arrows, Copia R24588; red boxes, Box38 elements; black boxes, MITE P580.2030; dark boxes, MITE G13554; shadow boxes, function unknown sequences; Blank arrows, marker genes. Chr, chromosomes. (**B**) Aliment of linker regions of stop codon to MITE P580.2030 (only displayed sequence logo). (**C**) Aliment of linker regions of MITE P580.2030 to Copia R24588. Blue box, simple sequence repeat of ‘A’. (**D**) Aliment of linker regions of Copia R24588 to initiation codon of NUDX1-1a. Red arrows, Box38. * indicated the pseudogenes which lack of full coding sequence.

Citronellol (dehydrogenation or oxidation) could transform from Nerol (cis-trans isomers of geraniol) and geraniol [26]. It seemed that geraniol is the basic version of high yNCG in cells. Moreover, geraniol production has been identified as a rose-specific pathway mediated by the expression of NUDX1-1a paralogs. We searched the candidate DNA markers based on the NUDX1-1a clusters of Oldbush, Rugosa, first haplotype of Zizhi (Zizhi_H1) and second haplotype of Zizhi (Zizhi_H2). According to the gene cluster on Chr2 of Oldbush (*RcNUDX1-1* 1st-5st copies), NUDX1-1a clusters of Rugosa (1st-6st copies), ZiZhi_H1 (1st-4st copies), and ZiZhi_H2 (1st-5st copies) were identified from the homologous chromosomes (Figure 2A and Figure S1). The ZiZhi_H2 5st and ZiZhi_H1 4st NUDX1-1a were two pseudogenes including early stop codons and their colinear *RcNUDX1-1* 5st copies were reported as pseudogenes. When ignoring the long regions between 2st and 3st copies (or 1st–2st copies of ZizhiH1), the synteny of NUDX1-1a clusters was conserved. Besides, the ‘Copia-NUDX1-1a-MITE’ or ‘MITE-Copia-NUDX1-1a’ were conserved blocks (excepted ZiZhi_H1 4st block lack Copia element). The 150 bp or 136 bp linker region of TGA (stop codon)-P580.2030 (MITE) was highly conserved (Figure 2B), while the linker region of P580.2030-Copia produced different indels, including a ‘A’-type simple sequence repeat (SSR-A) (Figure 2C).

BOX38 is a rose-specific cis-element produced by Copia. The sequence alignment indicated that the B38 element clustered with part overlapping repeats and its location was stable (Figure 2D). The first B38 overlapped with Copia (33-bp) and the last B38 located on 138 bp upstream ATG. Compared with four B38 repeats of Oldbush, 6–7 repeats of Rugosa, 5–6 repeats of Zizhi_H1, and six repeats of Zizhi_H2 indicated that the B38 repeats number was variable. Except for B38a and B38c, other B38 repeats (c1, c2, d2, f, e) with 1–2 SNPs were named and founded in Rugosa. Unlike the little information about MITE G13534 or unknown insert sequences, the promoter activity of B38 repeats was observed in Oldbush. This indicated that the expression of *NUDX1* relied on the interaction of potential trans-acting factors and B38 repeats. Considering that more repeat numbers would supply a stronger activation potential, we selected B38 as the candidate marker for further study.

### 3.3. Positively Correlation of Repeat Number of B38 and Essential Oil Yield

**Figure 3:**
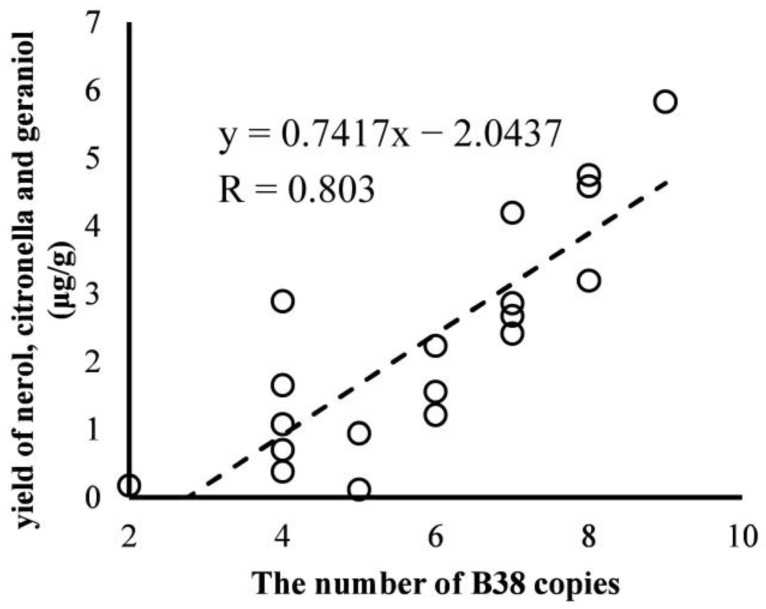
Correlation between the total yield of nerol, citronellol and geraniol and the repeat number of B38.

According to the PAGE of PCR products, the sequence length of B38 repeats regions and repeat number of B38 (RNB) were deduced (Table 1) based on sequence length/B38 length (38 bp or 34 bp). When RNB exceeded 4, the B38 length should be 34 bp as the overlap could not be neglected. Repeat number polymorphism was usually observed in different cultivars (even different individuals of same cultivar) and length polymorphism was only observed when RNB was 6. The RNB ranging from 2 to 9 showed a significant positive correlation with increasing yNCG (R = 0.803, *p* < 0.001) among 19 cultivars (Figure 3). When RNB was 4, it corresponded to four cultivars whose yNCG was differential. When RNB exceeded 6, the yNCG was higher than 2.414 μg/g. Together, this indicated that RNB was a positive correlation marker of yNCG.

## 4. Discussion

The perfume industry has been looking for roses with higher essential oil content to increase the essential oil yield and satisfy the market demand [8,17]. Over 18,000 modern roses have been generated by hybridization, breeding, or introgression since the 19th century, and some of them have a pleasant fragrance [5,29]. To identify and select roses with high essential oil, DNA marker which is associated with oil yield would be helpful. We compared the promoters, CDSs and TEs of NUDX1-1a cluster and found that most elements, including CDSs of all NUDX1-1a, Copia R24588, MITE P580.2030, and the linker region, were conserved. Microsatellite genotyping of *R. damascena* accessions from Europe possessed identical profiles [9]. Microsatellite seemed to not be a marker for the selection of high oil yield roses due to the narrow gene pool. A simple sequence repeat (SSR-A) was located on the linker region of P580.2030-Copia, while its polymorphism showed no correlation with oil yield (results of SSR polymorphism in 19 cultivars were no showed in text). Interestingly, polymorphism of a cis-element B38 repeats were associated with the yNCG, which is a key index of oil yield. Though there were exceptions (like high-oil-yield Russia with only 4 B38 repeats), the positive correlation of repeat number and the yNCG was obvious. Interestingly, when the two cultivars not belonging to Rugosa pedigree (Russia, *R. × centifolia* and Damask_Oil, *R. × damask*) were not counted, the correlation was more significant. This indicated the higher repeat number of B38 could be a selection criterion for oil roses in Rugosa pedigree. The higher repeat number of B38 contributed to the yNCG and seven repeats seemed to be a threshold for high oil yield. Besides, Plena_alba and Russia were intensively recommended by local managers of Pingyin City based on their sensual experience of more intense floral fragrance, though the two cultivars were not famous as Fenghua or Pingyin. Our study proved they were the top-two oil yield cultivars, which is contradictory to their Box38 repeats to some degree. Whether the patterns are appropriate for oil roses of another pedigree needs to be tested in more species, e.g., the famous Damask pedigree.

B38 repeats originated from 3′ long-terminal repeat (LTR) of Copia R24558 and the repeat number in promotor should tend to neutral evolution in wild species [24]. Seven repeats were also found in the wild Rugosa, which is the common ancestor of cultivars of Rugosa pedigree. Though Rugosa was a high-oil rose, its bush plant architecture was not applicable for cultivation [11]. Approximately 1300 years ago, flower growers of Pinying city selected the oil cultivars (namely Pinying) which stand erectly (Table S1). We found that it lost one repeat compared to Rugosa and still kept a high yNCG. Then, Plena with higher oil and its bud mutation Plena_alba were later selected and were the main cultivars in Pinyiing area until 1980s (Table S1). Other cultivars were mostly derived from the hybridization or introgression of Pinying, Plena, and other unknown parents, e.g., Fenghua and Zizhi, the local main cultivars now, which were selected by Pingyin Rose Research Institute in the 1980s. In the new breeding cultivars, a high number of B38 repeats (8–9) was maintained in the Rugosa background and selection of high oil seemed to be helpful in higher repeat numbers since only seven repeats were found in other rose species.

## 5. Conclusions

GC-MS analysis indicated that the main compounds (nerol, citronellol, and geraniol) represent an alternative index of essential oil yield in *Rosa × rugosa*. Genome comparison and analysis and polymorphic PCR detection showed that the repeat number of cis-element Box38 was significantly positively correlated to the essential oil yield. The essential oil yield of *Rosa × rugosa* cultivars was determined by the genetic background under the same environment and using the same oil extraction method. We suggest that the high number of B38 repeats (>6) should be an important selection standard for high-essential-oil roses in the similar climate regions under the same cultivation conditions. The B38-maker-selection could be a time-saving and efficient breeding tool for perfume roses.

## Figures and Tables

**Table 1:** B38 repeats polymorphism and oil yield of oil bearing roses.

Cultivars	Observed PCR Products #	Length of B38 Repeats	Repeat Number of B38	Yield (μg/g)
Xihu2	110	70	2	0.178
Daguo	184	144	4	0.387
Damask_Oil	184	144	4	0.701
Kushui	184	144	4	1.083
HENSA	184	144	4	1.656
Russia	184	144	4	2.892
Tuwei	202	162	5	0.117
Banchongban	202	162	5	0.951
Zizhi	235/240	195/200	6	1.218
Zizhi_DH	230/235	190/195	6	1.56
Pingyin	240	200	6	2.236
Liangyehong	265-270	225	7	2.414
Pingyin11	265	225	7	2.675
Plena_alba	265	225	7	2.863
Fenghua	265	225	7	3.195
Plena	290	250	8	4.588
Xihu3	290	250	8	4.753
Danbanhong	290	250	8	4.197
Xihu1	315	275	9	5.835

# Only the longest band was listed when 2 or more different bands were observed in replications.

## Data Availability

Not applicable.

## Supplementary Materials

The following supporting information can be downloaded at: https://www.mdpi.com/article/10.3390/biom13030439/s1, Figure S1: The synteny block of NUDX1-1a gene clusters of *R. rugosa* and *R. chinensis* by genome browser; Table S1: Accessions and oil yield of rose cultivars; Table S2: Percentage of organic compounds by GC-MS; Table S3: Main compounds of oil (μg per μL oil); Table S4: Yield of main compounds (μg per g flower).

## References

[1] Ainane T., Abdoul-Latif F.M., El Montassir Z., Attahar W., Ainane A., Giuffrè A.M. (2023). Essential oils rich in pulegone for insecticide purpose against legume bruchus species: Case of *Ziziphora hispanica* L. and *Mentha pulegium* L.. AIMS Agric. Food.

[2] Luan J., Yang M., Zhao Y., Zang Y., Zhang Z., Chen H. (2023). Aromatherapy with inhalation effectively alleviates the test anxiety of college students: A meta-analysis. Front. Psychol..

[3] Li J., Chen W., Liu H., Liu H., Xiang S., You F., Jiang Y., Lin J., Zhang D., Zheng C. (2023). Pharmacologic effects approach of essential oils and their components on respiratory diseases. J. Ethnopharmacol..

[4] Dekić M., Radulović N., Antonijević M., Dekić D., Ličina B. (2021). The essential oil of the condiment species *Clinopodium thymifolium* (Scop.) Kuntze: New natural products and seasonal variation. J. Sci. Food Agric..

[5] Raymond O., Gouzy J., Just J., Badouin H., Verdenaud M., Lemainque A., Vergne P., Moja S., Choisne N., Pont C. (2018). The Rosa genome provides new insights into the domestication of modern roses. Nat. Genet..

[6] Wissemann V., Roberts A., Debener T., Gudin S. (2003). Conventional Taxonomy of Wild Roses. Encyclopedia of Rose Science.

[7] Guterman I., Shalit M., Menda N., Piestun D., Dafny-Yelin M., Shalev G., Bar E., Davydov O., Ovadis M., Emanuel M. (2002). Rose scent genomics approach to discovering novel floral fragrance-related genes. Plant Cell.

[8] Kovacheva N., Rusanov K., Atanassov I. (2010). Industrial Cultivation of Oil Bearing Rose and Rose Oil Production in Bulgaria during 21ST Century, Directions and Challenges. Biotechnol. Biotechnol. Equip..

[9] Rusanov K., Kovacheva N., Vosman B., Zhang L., Rajapakse S., Atanassov A., Atanassov I. (2005). Microsatellite analysis of *Rosa damascena* Mill. accessions reveals genetic similarity between genotypes used for rose oil production and old Damask rose varieties. Theor. Appl. Genet..

[10] Cui W.-H., Du X.-Y., Zhong M.-C., Fang W., Suo Z.-Q., Wang D., Dong X., Jiang X.-D., Hu J.-Y. (2022). Complex and reticulate origin of edible roses (*Rosa*, Rosaceae) in China. Hortic. Res..

[11] Zang F., Ma Y., Tu X., Huang P., Wu Q., Li Z., Liu T., Lin F., Pei S., Zang D. (2021). A high-quality chromosome-level genome of wild *Rosa rugosa*. DNA Res..

[12] Şanli A., Karadoğan T. (2016). Geographical Impact on Essential Oil Composition of Endemic *Kundmannia Anatolica* Hub.-Mor.(Apiaceae). Afr. J. Tradit. Complement. Altern. Med..

[13] Gioffrè G., Ursino D., Labate M.L.C., Giuffrè A.M. (2020). The peel essential oil composition of bergamot fruit (*Citrus bergamia*, *Risso*) of Reggio Calabria (Italy): A review. Emir. J. Food Agric..

[14] Mendoza J.D.S., Correia L.C., Saad J.C.C., Siqueira W.J., Ming L.C., Campos F.G., Boaro C.S.F., Marques M.O.M. (2022). Effect of irrigation depth on biomass production and metabolic profile of *Lippia alba* (linalool chemotype) essential oil. Agric. Water Manag..

[15] Chatterjee S., Gupta S., Variyar P.S. (2015). Comparison of Essential Oils Obtained from Different Extraction Techniques as an Aid in Identifying Aroma Significant Compounds of Nutmeg (*Myristica Fragrans*). Nat. Prod. Commun..

[16] Liu Y., Zhi D., Wang X., Fei D., Zhang Z., Wu Z., Li Y., Chen P., Li H. (2018). Kushui Rose (*R. Setate x R. Rugosa*) decoction exerts antitumor effects in C. elegans by downregulating Ras/MAPK pathway and resisting oxidative stress. Int. J. Mol. Med..

[17] Xiao Z., Luo J., Niu Y., Wu M. (2018). Characterization of key aroma compounds from different rose essential oils using gas chromatography-mass spectrometry, gas chromatography–olfactometry and partial least squares regression. Nat. Prod. Res..

[18] Rusanov K.E., Kovacheva N.M., Atanassov I.I. (2011). Comparative GC/MS Analysis of Rose Flower and Distilled Oil Volatiles of The Oil Bearing Rose *Rosa damascena*. Biotechnol. Biotechnol. Equip..

[19] Spiller M., Berger R.G., Debener T. (2010). Genetic dissection of scent metabolic profiles in diploid rose populations. Theor. Appl. Genet..

[20] Venkatesha K., Gupta A., Rai A.N., Jambhulkar S., Bisht R., Padalia R.C. (2022). Recent developments, challenges, and opportunities in genetic improvement of essential oil-bearing rose (*Rosa damascena*): A review. Ind. Crop. Prod..

[21] Sun P., Schuurink R.C., Caissard J.-C., Hugueney P., Baudino S. (2016). My Way: Noncanonical Biosynthesis Pathways for Plant Volatiles. Trends Plant Sci..

[22] Tholl D., Gershenzon J. (2015). The flowering of a new scent pathway in rose. Science.

[23] Magnard J.-L., Roccia A., Caissard J.-C., Vergne P., Sun P., Hecquet R., Dubois A., Hibrand-Saint Oyant L., Jullien F., Nicolè F. (2015). Biosynthesis of monoterpene scent compounds in roses. Science.

[24] Conart C., Saclier N., Foucher F., Goubert C., Rius-Bony A., Paramita S.N., Moja S., Thouroude T., Douady C., Sun P. (2022). Duplication and Specialization of *NUDX1* in *Rosaceae* Led to Geraniol Production in Rose Petals. Mol. Biol. Evol..

[25] Sun P., Dégut C., Réty S., Caissard J., Hibrand-Saint Oyant L., Bony A., Paramita S.N., Conart C., Magnard J.L., Jeauffre J. (2020). Functional diversification in the *Nudix hydrolase* gene family drives sesquiterpene biosynthesis in *Rosa* × *wichurana*. Plant J..

[26] Feng L., Chen C., Li T., Wang M., Tao J., Zhao D., Sheng L. (2014). Flowery odor formation revealed by differential expression of monoterpene biosynthetic genes and monoterpene accumulation in rose (*Rosa rugosa* Thunb.). Plant Physiol. Biochem..

[27] Wang Y., Tang H., Debarry J.D., Tan X., Li J., Wang X., Lee T.-H., Jin H., Marler B., Guo H. (2012). *MCScanX*: A toolkit for detection and evolutionary analysis of gene synteny and collinearity. Nucleic Acids Res..

[28] Kearse M., Moir R., Wilson A., Stones-Havas S., Cheung M., Sturrock S., Buxton S., Cooper A., Markowitz S., Duran C. (2012). Geneious Basic: An integrated and extendable desktop software platform for the organization and analysis of sequence data. Bioinformatics.

[29] Hibrand Saint-Oyant L., Ruttink T., Hamama L., Kirov I., Lakhwani D., Zhou N.N., Bourke P.M., Daccord N., Leus L., Schulz D. (2018). A high-quality genome sequence of *Rosa chinensis* to elucidate ornamental traits. Nat. Plants.

