# Supplementary material for: BOX38, a DNA Marker for Selection of Essential Oil Yield of Rosa × rugosa
Source: Biomolecules. 2023 Feb 25;13(3):439. doi: 10.3390/biom13030439 (PMC10046031; doi:10.3390/biom13030439)
Supplement: Supplementary file 1 [file biomolecules-13-00439-s001.zip › Figure S1.pdf]

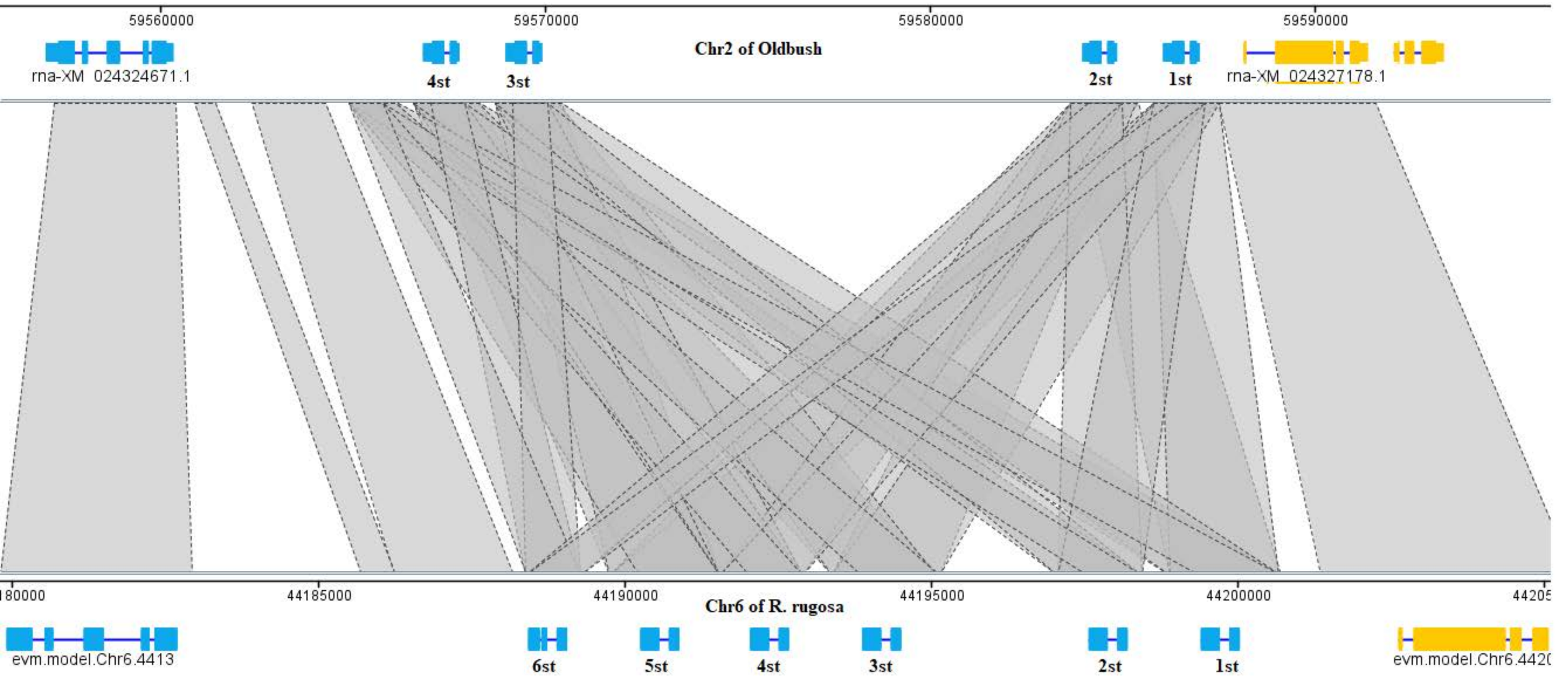

Figure S1. The synteny block of NUDX1-1a gene clusters of *R. rugosa* and *R. chinensis* by genome browser. 1-4st indicated 4 gene replications of *R. chinensis* and 1-6st for 6 replications of *R. rugosa*. The scaleplate indicated the location of genes on chromosomes.
